# Patient-Reported Outcomes and Revision Rates After ACL Reconstruction With Quadriceps Versus Hamstring and Patellar Tendon Autografts: Sex-Stratified Results From the Swedish Knee Ligament Registry

**DOI:** 10.1177/03635465251404876

**Published:** 2026-01-26

**Authors:** Dzan Rizvanovic, Markus Waldén, Magnus Forssblad, Riccardo Cristiani, Christoffer von Essen, Anders Stålman

**Affiliations:** † Department of Molecular Medicine and Surgery, Stockholm Sports Trauma Research Center, Karolinska Institutet, Stockholm, Sweden; ‡ Capio Artro Clinic, FIFA Medical Centre of Excellence, Stockholm, Sweden; § Unit of Public Health, Department of Health, Medicine and Caring Sciences, Linköping University, Linköping, Sweden; ‖ Capio Ortho Center Skåne, Malmö, Sweden; ¶ Ortopedi Stockholm, Stockholm, Sweden; Investigation performed at Stockholm Sports Trauma Research Center, Karolinska Institutet, Stockholm, Sweden

**Keywords:** ACLR, anterior cruciate ligament reconstruction, KOOS, Knee injury and Osteoarthritis Outcome Score, men, PRO, patient-reported outcome, graft, revision rate, women

## Abstract

**Background:**

Quadriceps tendon (QT) autografts are increasingly used worldwide in anterior cruciate ligament (ACL) reconstruction (ACLR). However, comparative outcome studies across graft types, particularly by sex, remain limited.

**Purpose:**

To compare patient-reported outcomes (PROs) and revision rates after primary ACLR with QT autografts in relation to patellar tendon (PT) and hamstring tendon (HT) autografts, stratified by sex.

**Study Design:**

Cohort study; Level of evidence, 3.

**Methods:**

Patients who underwent primary ACLR (2008-2022) were identified in the Swedish Knee Ligament Registry. The primary outcome was 2-year patient-reported knee function, assessed using the Knee injury and Osteoarthritis Outcome Score (KOOS). Clinical relevance was evaluated using the KOOS4 (mean of KOOS Pain, Symptoms, Sports/Recreation, and Quality of Life subscales), with thresholds for minimal important change (MIC, ≥9), patient acceptable symptom state (PASS, ≥79), and treatment failure (TF, ≤42). Adjusted logistic regression analyses assessed factors associated with MIC, PASS, and TF. The secondary outcome was 2-year revision ACLR, reported as rates and analyzed using Cox regression to estimate hazard ratios.

**Results:**

A total of 18,920 patients (44%) had 2-year KOOS data available. Women receiving QT grafts had a lower proportion of MICs achieved than those with HT grafts (61% vs 71%; *P* = .027). Among men, PASS was more frequently achieved with QT (51%) and HT grafts (48%) than with PT grafts (40%) (both *P*≤ .030). In the regression analyses, women with HT grafts had higher odds of achieving MIC (odds ratio [OR], 1.69 [95% CI, 1.19-2.42]; *P* = .004) and PASS (OR, 1.81 [95% CI, 1.28-2.58]; *P* < .001), and lower odds of TF (OR, 0.53 [95% CI, 0.31-0.88]; *P* = .015) compared with QT grafts. Additionally, no significant differences were observed between QT and PT grafts in women or among graft types in men.

Of 44,513 patients, 1019 (2.3%) underwent revision ACLR within 2 years: QT graft, 2.2% (28/1274); PT graft, 2.5% (50/2019); and HT graft, 2.3% (941/41,220) (*P* = .830). QT revision rates were 2.7% in women and 1.8% in men (*P* = .288). Graft type was not associated with revision hazard in adjusted Cox regression.

**Conclusion:**

QT autografts were associated with lower PROs compared with HT autografts in women, whereas no such differences were observed when compared with PT autografts or among men. Revision rates were similar across graft types, both overall and by sex.

Hamstring tendon (HT) and patellar tendon (PT) autografts are the most commonly used grafts in primary anterior cruciate ligament (ACL) reconstruction (ACLR).^33,49^ Quadriceps tendon (QT) autografts have, however, gained increasing attention as an alternative in recent years.^7,26,38,46^ QT grafts may offer favorable biomechanical properties, equivalent laxity, and lower or similar donor-site morbidity compared with both PT and HT grafts, although the use of QT grafts is associated with a short-term reduction in quadriceps strength.^15,17,26,32,45^

Previous studies—including observational studies, randomized controlled trials, and systematic reviews—have demonstrated no clinically significant differences in patient-reported knee function when comparing QT autografts with PT or HT autografts.^7,9,17,32,37,42,51,52^ However, recent registry data have consistently shown that female sex is associated with lower patient-reported outcomes (PROs) after ACLR.^6,12,35,37^ Potential explanations are likely multifactorial and may include greater postoperative laxity, reduced quadriceps strength, impaired neuromuscular control and landing mechanics, higher psychological distress, and lower likelihood of returning to preinjury sport among women.^1,8,11^ Notably, women appear more likely than men to receive HT rather than QT or PT autografts, although the factors driving this pattern remain unclear.^38^ These findings highlight an important knowledge gap regarding potential sex-specific differences in subjective outcomes and the influence of graft selection, underscoring the need for large-scale evaluations capable of addressing these interactions.

Revision ACLR is an important proxy for graft survival and treatment success, although existing studies report mixed findings when comparing revision rates between graft types.^5,7,24,27,41,51^

This study aimed to compare patient-reported knee function and revision ACLR rates after primary ACLR with QT autografts versus PT and HT autografts, stratified by sex, in a large national cohort. The hypothesis was that QT autografts would yield PROs and revision ACLR rates comparable to those of HT and PT autografts, but that outcomes may differ between female and male patients depending on graft type.

## Methods

Ethical approval for this study was obtained from the Swedish Ethical Review Authority (2022-06852-02), and the study was also approved by the steering committee of the Swedish Knee Ligament Registry (SKLR) (2025-06-24).

Prospectively collected SKLR data were used to conduct a registry-based cohort study. The SKLR, established in 2005, accounts for >90% of all ACLRs performed in Sweden each year.^22^ The registry contains pre-, intra-, and postoperative information reported by surgeons and patients. Surgeon-reported variables include the date of injury and surgery, activity at the time of injury, associated injuries, and surgical details such as graft type, treatment of associated injuries, and operative time. Patients are asked to complete the Knee injury and Osteoarthritis Outcome Score (KOOS) questionnaire preoperatively and at 1, 2, 5, and 10 years postoperatively. Subsequent surgeries—including revision or contralateral ACLR—are recorded separately and linked to the index procedure.

### Study Population

The registry included all primary and revision ACLRs registered in the SKLR between 2005 and 2024. Patients undergoing primary index ACLR between January 1, 2008, and December 31, 2022, were assessed for eligibility. To account for the registry's start-up period, patients who underwent surgery between 2005 and 2007 were excluded. To ensure a complete 2-year follow-up, only patients who underwent surgery on or before December 31, 2022, were included. Contralateral and revision ACLRs were excluded. Further exclusion criteria were age <16 years due to differences in treatment strategy and graft selection in skeletally immature patients, posterior cruciate ligament (PCL) injuries, concomitant ligament injuries requiring surgical treatment—including medial collateral ligament [(MCL), lateral collateral ligament, or posterolateral ligament complex injuries, fractures, tendon, vascular, or nerve injuries, use of graft types other than QT, PT, or HT autografts, missing graft information, and missing surgeon code.

After applying these criteria, 2 study cohorts were analyzed: (1) the KOOS cohort, consisting of all patients with available 2-year KOOS data after primary ACLR who had not undergone revision or contralateral ACLR within 2 years, used for analyses of PROs; and (2) the revision cohort, comprising all patients who underwent primary ACLR, from whom those with a revision ACLR within 2 years were identified and analyzed based on characteristics and surgical details from their primary procedure, used for analyses of revision ACLR.

### Exposure and Outcome

Graft type was the primary exposure, categorized as QT, PT, or HT autograft, as reported in the SKLR. QT autografts were performed with or without a bone block; however, bone block use was not recorded until 2018, and was unspecified in nearly half of the cases. Therefore, all QT grafts were analyzed together irrespective of bone block use. The primary outcome was patient-reported knee function 2 years after primary ACLR, assessed using the KOOS. Analyses included KOOS4, calculated as the mean of the Pain, Symptoms, Sports/Recreation (Sports/Rec), and knee-related Quality of Life (QoL) subscales, to facilitate interpretation and improve clinical relevance by excluding the less relevant Activities of Daily Living (ADL) subscale in this population.^4^ In addition, all individual KOOS subscales (Pain, Symptoms, ADL, Sports/Rec, and QoL) were analyzed.^4,40^ Threshold values for minimal important change (MIC, ≥9), patient acceptable symptom state (PASS, ≥ 79), and treatment failure (TF, ≤ 42) were applied to the KOOS4 (mean of the KOOS Pain, Symptoms, ADL, Sports/Rec, and QoL subscales) to evaluate clinically relevant differences between the graft groups.^39^ These thresholds were derived from a similar patient population.^18,19,39^ The secondary outcome was revision ACLR within 2 years of the primary index procedure.

### Statistical Analyses

All statistical analyses were performed using SPSS Statistics Version 29 (IBM Corp). Continuous variables were presented as median (25th-75th percentile) or mean (standard deviation) and compared using the Kruskal-Wallis test or 1-way analysis of variance (ANOVA), respectively. Categorical variables were presented as number (percentage) and compared using the chi-square test. Pairwise comparisons were performed using Mann-Whitney *U* tests for non-normally distributed continuous variables, Bonferroni-adjusted post hoc tests after 1-way ANOVA for normally distributed continuous variables, or chi-square tests for categorical variables.

Adjusted multivariable logistic regression analyses were used to assess the association between graft type and the achievement of MIC, PASS, and TF on the KOOS4, with sex-stratified subgroup analyses performed to evaluate whether these associations were consistent across sexes. The logistic regression models of MIC, PASS, and TF were internally validated using c-statistics (discrimination) and the Hosmer–Lemeshow test (calibration). Cox proportional hazards regression analysis was used to evaluate the association between graft type and revision ACLR within 2 years of the primary surgery. Results were reported as odds ratios (ORs) or hazard ratios with 95% CIs. Age at surgery (≤20, 21-30, 31-40, and >40 years) and patient sex were first entered into the regression models. The following variables were then introduced using a forward stepwise method and retained in the model if *P* < .10: pivoting sport injury (yes/no), MCL injury (yes/no), meniscal injury (yes/no), cartilage injury (yes/no), time from injury to surgery (continuous), preoperative KOOS4 (continuous), clinic volume (low/high), surgeon volume (low/high), year of surgery (continuous), and ACL graft type (QT, PT, or HT).^37^ Pivoting sports included soccer, floorball, handball, ice hockey, American football, rugby, and basketball. Surgeons and clinics were classified based on their previous and current surgical volumes. The cutoffs were determined from previous SKLR studies.^37,38^ Surgeons were classified as high-volume if they had performed ≥50 ACLRs in total and ≥29 annually.^37,38^ Clinics were considered high-volume if they had ≥500 total cases and an annual volume of ≥56 ACLRs.^37,38^ Surgeons and clinics that did not meet these thresholds were classified as low-volume. Statistical significance was defined as *P* < .05 (2-tailed) in all analyses.

## Results

### KOOS Cohort

**Figure 1.**
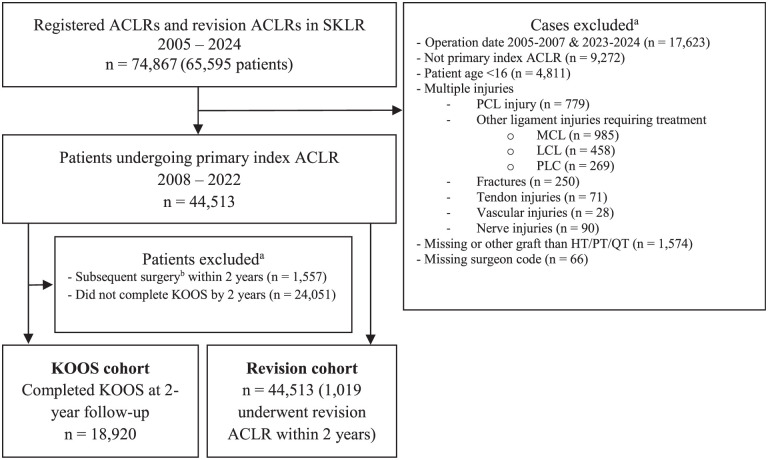
Flowchart of patient selection. ACLR, anterior cruciate ligament reconstruction; HT, hamstring tendon; KOOS, knee injury and osteoarthritis outcome score; LCL, lateral collateral ligament; MCL, medial collateral ligament; PCL, posterior cruciate ligament; PLC, posterolateral complex; PT, patellar tendon; SKLR, Swedish Knee Ligament Registry; QT, quadriceps tendon. *^a^* Patients may appear in several groups. *^b^* Subsequent surgery = revision or contralateral ACLR.

A total of 18,920 patients (44%) completed the 2-year KOOS without undergoing revision or contralateral ACLR during follow-up (Figure 1).

The median age at surgery was 27 years (25th-75th percentile, 20-39 years), and 50.2% were women. Patient characteristics by graft type for the KOOS cohort are presented in Table 1. Statistically significant differences were observed for age, associated injuries, time to surgery, and operating time.

**Table 1:** Patient Characteristics at Primary ACLR by Graft Type in Patients With 2-Year KOOS Follow-up*^*a*^*

	QT	PT	HT	*P*
Patients, n	425	803	17,692	
Age at surgery	27 (19-38)	25 (19-38)	27 (20-39)	**.003**
Sex				.150
Female	200 (47.1)	384 (47.8)	8918 (50.4)	
Male	225 (52.9)	419 (52.2)	8774 (49.6)	
BMI, kg/m^2^	24.6 (22.4-27.1)	24.5 (22.8-26.9)	24.4 (22.5-26.5)	.149
Pivoting sports injury	213 (50.1)	9,424 (53.3)	431 (53.7)	.418
MCL injury	62 (14.6)	113 (14.1)	574 (3.2)	**<.001**
Meniscal injury	226 (53.2)	351 (43.7)	8074 (45.6)	**.004**
Cartilage injury	126 (29.6)	183 (22.8)	5026 (28.4)	**.002**
Time to surgery, months	6 (3-13)	7 (3-14)	8 (4-18)	**<.001**
Operating time, min	90 (75-104)	80 (65-95)	66 (54-85)	**<.001**
ACLR by high-volume surgeon	369 (86.8)	517 (64.4)	11,298 (63.9)	**<.001**
ACLR by high-volume clinic	269 (63.3)	483 (60.1)	8911 (50.4)	**<.001**

a Data are reported as median (25^th^-75^th^ percentile) or n (%). Bold *P* values indicate statistical significance. Missing patient values: activity at time of injury, n = 8; BMI, n = 5349; time to surgery, n = 393; and operating time, n = 851. ACLR, anterior cruciate ligament reconstruction; BMI, body mass index; HT, hamstring tendon; high-volume surgeon, ≥50 ACLRs in total and ≥29 ACLRs annually; high-volume clinic, ≥500 ACLRs in total and ≥56 ACLRs annually; KOOS, Knee injury and Osteoarthritis Outcome Score; MCL, medial collateral ligament; PT, patellar tendon; QT, quadriceps tendon.

**Figure 2.**
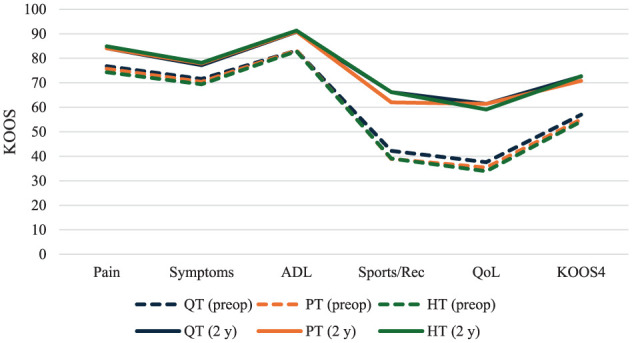
KOOS subscales by graft type preoperatively and 2 years after primary ACLR. KOOS4 scores and KOOS4 for QT, PT, and HT grafts. Range 0 to 100, with higher scores indicating better outcomes. ACLR, anterior cruciate ligament reconstruction; ADL, Activities of Daily Living; HT, hamstring tendon; KOOS, Knee injury and Osteoarthritis Outcome Score; KOOS4, mean of the Pain, Symptoms, ADL, Sports/Rec, and QoL subscales; QoL, Quality of Life; QT, quadriceps tendon; PT, patellar tendon; Sports/Rec, Sports/Recreation.

KOOS subscale scores improved from preoperatively to 2 years across all graft types (Figure 2). Preoperatively, patients receiving QT grafts demonstrated scores similar to or slightly higher than those of PT and HT across all subscales. Significant preoperative differences were observed for Pain (*P* = .007), ADL (*P* = .032), QoL (*P* < .001), and KOOS4 (*P* = .008), with Bonferroni-adjusted pairwise comparisons indicating differences between QT and HT grafts in Pain (*P* = .032), ADL (*P* = .027), QoL (*P* = .001), and KOOS4 (*P* = .011). At 2-year follow-up, mean KOOS scores were similar across graft types, with small but statistically significant differences in Sports/Rec (*P* < .001), QoL (*P* = .026), and KOOS4 (*P* = .025). QT and HT autografts had slightly higher Sports/Rec scores than PT autografts (QT vs PT, *P* = .035; HT vs PT, *P* < .001), and HT autografts also showed slightly higher KOOS4 scores compared with PT autografts (*P* = .021). However, HT autografts had marginally lower QoL scores than PT autografts (*P* = .021).

Stratified by sex, small differences in mean KOOS4 scores were observed. Among women, the mean preoperative KOOS4 was 55 (SD, 19) for QT, 56 (SD, 19) for PT, and 52 (SD, 18) for HT (*P* = .006) grafts, with a significant pairwise difference between PT and HT grafts (*P* = .017, Bonferroni-adjusted). Among male patients, preoperative KOOS4 was 59 (SD, 19) for QT, 55 (SD, 19) for PT, and 56 (SD, 18) for HT grafts (*P* = .032), with a significant pairwise difference between QT and PT grafts (*P* = .027, Bonferroni-adjusted). At 2-year follow-up, KOOS4 among women did not differ significantly by graft type: QT, 70 (SD, 20); PT, 71 (SD, 18); and HT, 72 (SD, 19) (*P* = .336). Among men, KOOS4 at 2 years was 75 (SD, 20) for QT; 71 (SD, 20) for PT; and 74 (SD, 19) for HT (*P* = .003), with significant pairwise differences between QT and PT grafts (*P* = .041) and between PT and HT grafts (*P* = .003, Bonferroni-adjusted).

**Figure 3.**
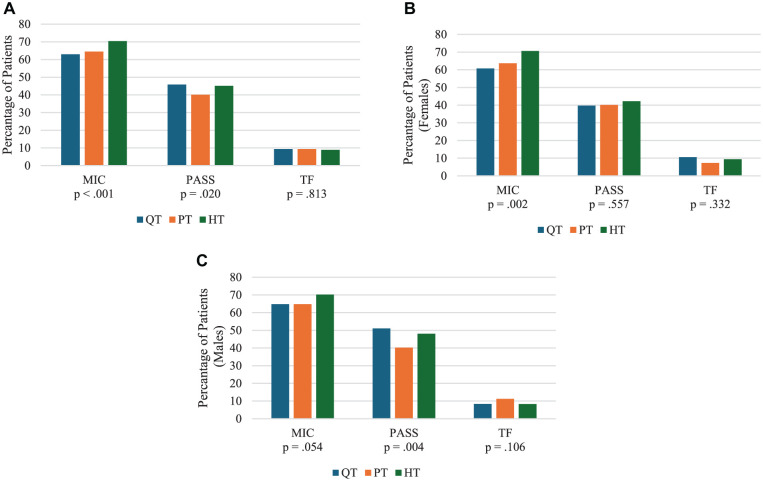
(A) Percentage of patients achieving MIC, PASS, and TF on the KOOS4 by graft type at 2-year follow-up. Statistically significant pairwise comparisons (Bonferroni-adjusted) were observed: QT vs HT (MIC, *P* = .009), and PT versus HT (MIC, *P* = .006; PASS, *P* = .018). (B) Percentage of female patients achieving MIC, PASS, and TF on the KOOS4 by graft type at 2-year follow-up. A statistically significant pairwise comparison (Bonferroni-adjusted) was observed: QT versus HT (MIC, *P* = .027). (C) Percentage of male patients achieving MIC, PASS, and TF on the KOOS4 by graft type at 2-year follow-up. Statistically significant pairwise comparisons (Bonferroni-adjusted) were observed: QT vs PT (PASS, *P* = .030) and PT versus HT (PASS, *P* = .006). HT, hamstring tendon; KOOS4, mean score of the Pain, Symptoms, Sports/Recreation, and Quality of Life subscales; MIC, minimal important change; PASS, patient acceptable symptom state; PT, patellar tendon; QT, quadriceps tendon; TF, treatment failure.

The proportion of patients achieving MIC, PASS, and TF at 2 years differed by graft type (Figure 3A). MIC was less often achieved with QT and PT grafts compared with HT grafts, and PASS was less frequent with PT grafts than with HT grafts. Among women, a lower proportion achieved MIC with QT grafts than with HT grafts (Figure 3B), whereas among men, a higher proportion achieved PASS with QT and HT grafts than with PT grafts (Figure 3C).

In the adjusted logistic regression analyses, QT grafts were associated with worse outcomes compared with HT grafts (all, *P*≤ .006), but not with PT grafts (Table 2). The MIC, PASS, and TF models had c-statistics of 0.71 (95% CI, 0.70-0.72), 0.70 (95% CI, 0.69-0.71), and 0.76 (95% CI, 0.74-0.77), respectively. Hosmer-Lemeshow tests indicated no evidence of lack of fit (χ^2^ = 6.44-10.05; *df* = 6; all, *P* > .10). Patients undergoing ACLR with HT grafts had 42% higher odds of achieving MIC (OR, 1.42 [95% CI, 1.11-1.81]; *P* = .005), 43% higher odds of reaching PASS (OR, 1.43 [95% CI, 1.13-1.81]; *P* = .003), and 41% lower odds of experiencing TF (OR, 0.59 [95% CI, 0.41-0.86]; *P* = .006) compared with QT grafts. However, subanalyses revealed that differences in odds of achieving MIC, PASS, and TF between QT and HT grafts were only observed in female patients (all, *P*≤ .015), with no significant associations found in men (Table 3).

**Table 2:** Adjusted Logistic Regression Results for Factors Influencing the Odds of Achieving MIC, PASS, and TF KOOS4 Within 2 Years After Primary ACLR*^*a*^*

	MIC, n = 13,772		PASS, n = 13,772		TF, n = 13,772	
Variable	OR (95% CI)	*P*	OR (95% CI)	*P*	OR (95% CI)	*P*
Age at surgery, years						
≤20	Ref		Ref		Ref	
21-30	1.19 (1.08-1.31)	**<.001**	1.15 (1.05-1.27)	**.003**	0.77 (0.66-0.91)	**.003**
31-40	1.50 (1.34-1.69)	**<.001**	1.59 (1.43-1.77)	**<.001**	0.71 (0.58-0.85)	**<.001**
>40	2.11 (1.87-2.39)	**<.001**	2.04 (1.83-2.28)	**<.001**	0.58 (0.48-0.71)	**<.001**
Sex						
Female	0.88 (0.81-0.95)	**.002**	0.88 (0.82-0.95)	**<.001**	0.98 (0.86-1.12)	.802
Male	Ref		Ref		Ref	
Meniscal injury	–		–		0.87 (0.76-0.99)	**.034**
Cartilage injury	0.80 (0.73-0.87)	**<.001**	0.81 (0.75-0.88)	**<.001**	1.27 (1.10-1.47)	**<.001**
Year of surgery	1.02 (1.01-1.03)	**<.001**	1.02 (1.01-1.03)	**<.001**	0.98 (0.96-0.99)	**.002**
Surgeon volume						
Low	0.87 (0.80-0.94)	**<.001**	0.87 (0.80-0.94)	**<.001**	–	
High	Ref		Ref		Ref	
Preop KOOS4	0.96 (0.96-0.96)	**<.001**	1.04 (1.04-1.04)	**<.001**	0.95 (0.94-0.95)	**<.001**
ACL graft						
QT	Ref		Ref		Ref	
PT	1.03 (0.76-1.39)	.844	1.02 (0.77-1.37)	.876	0.76 (0.48-1.21)	.247
HT	1.42 (1.11-1.81)	**.005**	1.43 (1.13-1.81)	**.003**	0.59 (0.41-0.86)	**.006**

a All values are adjusted for the included variables in each model and for pivoting sport injury, MCL injury, time from injury to surgery, and clinic volume. Bold *P* values indicate statistical significance. ACL, anterior cruciate ligament; ACLR, anterior cruciate ligament reconstruction; HT, hamstring tendon; KOOS4, mean score of the Pain, Symptoms, Sports/Recreation, and Quality of Life subscales; MCL, medial collateral ligament injury; MIC, minimal important change; OR, odds ratio; PT, patellar tendon; PASS, patient acceptable symptom state; Preop, preoperatively; QT, quadriceps tendon; Ref, reference; TF, treatment failure.

**Table 3:** Adjusted Logistic Regression Results for Graft Influence on Achieving MIC, PASS, and TF Within 2 Years After Primary ACLR in Female and Male Patients*^*a*^*

	MIC		PASS		TF	
	OR (95% CI)	*P*	OR (95% CI)	*P*	OR (95% CI)	*P*
Women, n = 6896						
QT	Ref		Ref		Ref	
PT	1.24 (0.80-1.93)	.342	1.52 (0.98-2.34)	.059	0.59 (0.30-1.18)	.135
HT	1.69 (1.19-2.42)	**.004**	1.81 (1.28-2.58)	**<.001**	0.53 (0.31-0.88)	**.015**
Men, n = 6630						
QT	Ref		Ref		Ref	
PT	0.82 (0.54-1.25)	.352	0.73 (0.49-1.08)	.117	0.95 (0.49-1.83)	.872
HT	1.20 (0.86-1.69)	.290	1.16 (0.84-1.61)	.368	0.62 (0.36-1.08)	.090

a All analyses are adjusted for age at surgery, pivoting sport injury, MCL injury, meniscal injury, cartilage injury, time from injury to surgery, surgeon volume, clinic volume, year of surgery, and preoperative KOOS4. The bold *P* value indicates statistical significance. ACLR, anterior cruciate ligament reconstruction; HT, hamstring tendon; KOOS4, mean score of the Pain, Symptoms, Sports/Recreation, and Quality of Life subscales; MIC, minimal important change; OR, odds ratio; PASS, patient acceptable symptom state; PT, patellar tendon; QT, quadriceps tendon; Ref, reference; TF, treatment failure.

Patient characteristics differed between KOOS responders and nonresponders (Table 4). Nonresponders were slightly younger, more often men, and a higher proportion sustained their ACL injury during pivoting sports (all, *P* < .001). The proportion of responders varied by graft type: 34.8% for QT, 41.7% for PT, and 44.5% for HT (*P* < .001).

**Table 4:** Patient Characteristics at Primary ACLR: Responders Versus Nonresponders*^*a*^*

	Responders	Nonresponders	*P*
Patients, n	18,920	24,013	
Age at surgery	27 (20-39)	25 (20-34)	**<.001**
Sex			**<.001**
Female	9502 (50.2)	8680 (36.1)	
Male	9418 (49.8)	15,333 (63.9)	
BMI, kg/m^2^	24.4 (22.5-26.6)	24.5 (22.6-26.8)	**.004**
Pivoting sports injury	10,068 (53.2)	14,063 (58.6)	**<.001**
MCL injury	749 (4.0)	925 (3.9)	.581
Meniscal injury	8651 (45.7)	11,423 (47.6)	**<.001**
Cartilage injury	5335 (28.2)	6115 (25.5)	**<.001**
Time to surgery, months	8 (4-18)	8 (4-18)	.235
Operating time, min	68 (55-86)	69 (55-88)	.114
ACL graft			**<.001**
QT	425 (2.2)	798 (3.3)	
PT	803 (4.2)	1124 (4.7)	
HT	17,692 (93.5)	22,091 (92)	

a Data are reported as median (25^th^-75^th^ percentile) or n (%). Bold *P* values indicate statistical significance. ACL, anterior cruciate ligament; ACLR, anterior cruciate ligament reconstruction; BMI, body mass index; HT, hamstring tendon; MCL, medial collateral ligament; PT, patellar tendon; QT, quadriceps tendon.

### Revision Cohort

Revision ACLR within 2 years of the primary surgery was performed in 1019 of 44,513 patients (2.3%) (Figure 1). Patient characteristics at the time of primary ACLR by graft type among patients who later underwent revision within 2 years are presented in Table 5. There were no significant differences in revision ACLR rates between graft types: 2.2% (28/1274) for QT, 2.5% (50/2019) for PT, and 2.3% (941/41,220) for HT (*P* = .830).

**Table 5:** Patient Characteristics at Primary ACLR by Graft Type Among Patients Undergoing Revision ACLR Within 2 Years*^*a*^*

	QT	PT	HT	*P*
Patients, n	28 (2.2)	50 (2.5)	941 (2.3)	.830
Age at surgery, years	22 (18-25)	20 (17-26)	20 (18-26)	.968
Sex				.373
Female	14 (50)	33 (66)	552 (58.7)	
Male	14 (50)	17 (34)	389 (41.3)	
BMI, kg/m^2^	23.1 (21.9-26.2)	24.1 (22.5-25.9)	24.2 (21.0-26.1)	.585
Pivoting sports injury	21 (75)	45 (90)	685 (72.8)	**.026**
MCL injury	2 (7.1)	5 (10)	40 (4.3)	.136
Meniscal injury	14 (50)	22 (44)	431 (45.8)	.876
Cartilage injury	3 (10.7)	7 (14)	195 (20.7)	.232
Time to surgery, months	5 (3-7)	4 (3-8)	3 (2-6)	**.046**
Operating time, min	88 (71-99)	76 (68-91)	65 (55-85)	**<.001**

a Data are reported as median (25^th^-75^th^ percentile) or n (%). Bold *P* values indicate statistical significance. Missing patient values: BMI, n = 398; time to surgery, n = 14; and operating time, n = 47. No significant differences in revision rates were found across graft types when analyzed by sex. ACLR, anterior cruciate ligament reconstruction; BMI, body mass index; HT, hamstring tendon; MCL, medial collateral ligament; PT, patellar tendon; QT, quadriceps tendon.

Among women, revision rates were 2.7% (14/513) for QT, 2.1% (17/804) for PT, and 2.2% (390/17,544) for HT grafts (*P* = .727). Among men, the rates were 1.8% (14/761) for QT, 2.7% (33/1215) for PT, and 2.3% (554/23,676) for HT grafts (*P* = .453). Revision rates did not differ significantly between women and men with QT grafts (2.7% vs 1.8%; *P* = .288).

In the Cox regression analysis, graft type was not associated with the hazard of revision ACLR within 2 years (Table 6). Instead, younger age, male sex, pivoting sport injury, MCL injury, high clinic volume, and lower preoperative KOOS4 were associated with an increased hazard of revision.

**Table 6:** Cox Regression Analysis of Factors Influencing the Hazard of Revision ACLR Within 2 Years*^a^*

Variable	HR (95% CI)	*P*
Age at surgery, years		
≤20	Ref	
21-30	0.53 (0.45-0.62)	**<.001**
31-40	0.26 (0.20-0.34)	**<.001**
>40	0.20 (0.14-0.28)	**<.001**
Sex		
Female	0.83 (0.71-0.96)	**.015**
Male	Ref	
Pivoting sports injury	1.50 (1.26-1.79)	**<.001**
MCL injury	1.55 (1.11-2.16)	**.010**
Clinic volume		
Low	0.76 (0.66-0.88)	**<.001**
High	Ref	
Preop KOOS4	0.99 (0.99-1)	**.003**
ACL graft	–	.989

a All values are adjusted for variables in the model—including meniscal injury, cartilage injury, time from injury to surgery, year of surgery, and surgeon volume (n = 30,769; 741 revision ACLR within 2 years). The bold *P* value indicates statistical significance. ACL, anterior cruciate ligament; ACLR, anterior cruciate ligament reconstruction; HR, hazard ratio; KOOS4, mean score of the Pain, Symptoms, Sports/Recreation, and Quality of Life subscales; MCL, medial collateral ligament; Preop, preoperative; Ref, reference.

## Discussion

The key finding of this study was that, among female patients, QT autografts were associated with inferior clinical improvement, lower patient satisfaction, and increased treatment failure compared with HT autografts, but not with PT autografts. Conversely, in male patients, QT autografts had PROs similar to those of HT and PT autografts. Despite the variation in patient-reported knee function, revision ACLR rates within 2 years did not differ significantly between graft types, both overall and when stratified by sex.

This study demonstrated significantly greater odds of achieving MIC and PASS, and lower odds of TF among patients receiving HT grafts compared with those receiving QT grafts. When stratified by sex, female patients receiving HT grafts had 69% higher odds of achieving MIC, 81% higher odds of attaining PASS, and 47% lower odds of treatment failure compared with those receiving QT grafts. No significant differences were observed between QT and PT in women, nor among men across graft types in the adjusted logistic regression analyses. While the primary focus of this study was the comparison of QT with the other graft types, comparisons between HT and PT grafts revealed a higher proportion of patients achieving MIC and PASS with HT grafts. While similar findings have previously been reported by other large cohort studies,^6,37^ others have found no significant differences in PROs between HT and PT grafts.^23^ Taken together, these results suggest that graft selection may influence PROs after ACLR, and that this association may vary by sex.

However, several previous studies have reported comparable PROs between QT autografts and PT or HT autografts after ACLR.^7,9,14,17,26,32,42,51,52^ Several factors may explain the apparent difference observed in this study compared with these previous reports. First, there is considerable heterogeneity in outcome measures across studies, and those incorporating the KOOS have primarily relied on mean scores,^3,14,26,51,52^ which may fail to identify clinically relevant differences otherwise captured with MIC, PASS, and TF,^39^ as shown in the present study. Second, although HT autografts predominated, the present study included the largest national multicenter cohort to date, derived from the SKLR, allowing for the detection of subtle but potentially meaningful differences between graft types, particularly in sex-stratified analyses that smaller studies may not reveal.^3,14,42,52^ Third, the regression models were adjusted for a comprehensive set of potential confounders, which may partly explain discrepancies from earlier research that did not account for differences in patient and/or surgical characteristics influencing PROs by graft type.^3,14,42,51,52^ For example, surgical volume has been shown to affect both graft choice and PROs, with patients operated on by high-volume surgeons having increased odds of receiving QT or PT grafts compared with HT grafts and experiencing greater improvement and satisfaction after primary ACLR.^37,38^ In the Swedish setting, QT grafts are most often performed by experienced surgeons, suggesting that technical errors due to inexperience are likely limited.^38^ To address potential confounding by surgical experience, both surgeon and clinic volume were accounted for in the present analyses. Although HT autografts were most common, this reflects national practice patterns, and adjustment for relevant confounders in the regression analyses reduced the likelihood that graft predominance biased the patient-reported results. Finally, although female sex has been associated with lower PROs in general after ACLR,^6,12,35,37^ very limited research has examined PROs stratified by sex when comparing QT with PT or HT autografts. One study found no sex differences in subjective function across graft types but reported delayed physical recovery (strength and hop symmetry) in women receiving QT autografts compared with HT autografts at 1 year postoperatively, a result not observed in men.^10^ Another study similarly noted delayed quadriceps recovery and a higher rate of early extension deficits in females after QT ACLR.^16^ These findings suggest that rehabilitation response may differ by sex, particularly following QT graft harvesting, and could contribute to the inferior PROs observed in female patients in the present study. The current results contribute to identifying potential sex-specific differences in graft selection, highlighting the importance of individualized treatment strategies.

### Revision ACLR

The revision ACLR rates within 2 years of primary surgery did not differ significantly between QT (2.2%), PT (2.5%), and HT (2.3%) autografts, and graft type was not a significant factor in the adjusted analysis. Consistent with previous studies, the QT autograft demonstrated graft survival rates comparable to those of PT and HT autografts.^7,14,17,32,50^

However, contrasting evidence exists. A Danish registry study reported higher revision rates for QT autografts (4.7%) compared with PT (1.5%) and HT autografts (2.3%) at 2 years,^27^ although subsequent analyses suggested that these findings were largely explained by a learning curve in QT graft usage,^24^ a factor that was partly accounted for in the present study through adjustment for both annual and total surgeon and clinic ACLR volume. Interestingly, in the present study, low-volume clinics were found to have lower odds of revision ACLR, which may reflect selection bias related to differences in patient characteristics (eg, activity level or injury complexity) or a greater tendency among high-volume centers to proceed with revision surgery when indicated, rather than true differences in surgical failure rates. Further, a Norwegian registry analysis found a 2-year revision rate of 3.6% for QT, 2.5% for HT, and 1.2% for PT grafts, with PT grafts showing a significantly lower revision risk than QT grafts in adjusted analyses.^51^ On the contrary, lower revision rates were observed with QT autografts compared with HT autografts (2.8% vs 4.9%, OR 2.7) in a recent cohort study.^41^ The variability in findings across studies, including those from national registries, highlights the potential for confounding by treatment. Graft choice is often guided by surgeon preference,^44^ and in Sweden, HT has been the predominant graft, while QT or PT grafts may be more frequently selected when surgeons anticipate a higher reinjury risk or for specific indications, such as concomitant MCL injury.^38^ This selection pattern, together with the relatively small number of revisions, may partly explain the absence of significant differences in revision rates, despite some previous studies reporting higher revision risks for HT autografts compared with PT.^23,29,31^ These factors limit causal inference regarding graft performance and underscore the need for controlled studies to better isolate the effect of graft choice on revision outcomes.

While female sex has been reported as a risk factor for primary ACL injury,^30^ the evidence regarding sex-related differences in graft rupture and revision ACLR remains conflicting.^2,5,28,47,48^ Most studies have focused on PT and HT autografts: some have reported a higher revision risk in male patients,^2,28^ whereas others have found no association between sex and revision risk,^5,20,43,47^ or even a slightly increased risk in women.^48^ Studies including QT autografts have not specifically analyzed revision rates stratified by sex.^7,14,17,27,32,51^ In this study, women had an adjusted 17% lower hazard of revision compared with men; however, no statistically significant sex-specific differences between graft types were observed within 2 years. Revision rates for QT autografts were 2.7% in women versus 1.8% in men, and the clinical significance of this difference in long-term graft survival remains uncertain and warrants further investigation.

### Limitations

This study has limitations inherent to its registry-based design. Several potentially important factors were not captured—including patient activity level, rationale for graft selection, rehabilitation protocols, graft failures that did not result in revision ACLR surgery, and specific indications for revision ACLR. Differences in surgical technique were also not accounted for—including whether QT grafts were harvested with or without a bone block or as partial- versus full-thickness autografts. However, recent evidence suggests no significant differences in revision rates or PROs between soft-tissue QT and QT with a bone block or between partial- and full-thickness QT autografts.^21,25^ The analyses of MIC, PASS, and TF were limited to patients with available KOOS data at 2 years. While the response rate in the SKLR (44%) is higher than in other Scandinavian ACL registries (41% in NKLR at 2 years, 25% in DKLR at 1 year),^24,51^ the potential for nonresponse bias cannot be excluded. Responders and nonresponders differed in sex distribution and showed slight differences in age, graft type, and injury characteristics. These differences may have influenced the observed associations, potentially accentuating sex-related effects or failing to capture similar patterns among men who did not respond. Furthermore, given the lower response rate among QT recipients compared with the HT group, there may be some uncertainty in the QT estimates. Notably, previous studies have shown no clinically meaningful differences in KOOS between responders and nonresponders, suggesting that the impact of nonresponse bias on the present findings is most likely low.^34,36^ Moreover, all relevant baseline variables were included as covariates in the regression models to minimize the influence of such differences. While the overall registry cohort was large and included the largest number of patients who had QT revisions to date among comparable studies, the number of QT revisions within 2 years was low (28/1,274), introducing a risk of sparse-data bias and reducing statistical power for between-graft and sex-stratified comparisons. Accordingly, the absence of statistically significant differences in revision rates or in the Cox regression analysis should be interpreted with caution. Finally, the KOOS and other PRO measures have been questioned regarding content validity, particularly the ADL subscale in younger patients.^13^ To address this, KOOS4 was used, excluding the least relevant subscale, to improve clinical relevance in this population.^4^

## Conclusion

QT autografts were associated with lower PROs than HT autografts in women at 2 years, whereas no such differences were observed with PT autografts or among men. Despite these differences in PROs, 2-year revision rates were similar across graft types, both overall and by sex.

## References

[1] Bruder AM Culvenor AG King MG , et al. Let’s talk about sex (and gender) after ACL injury: a systematic review and meta-analysis of self-reported activity and knee-related outcomes. Br J Sports Med. 2023;57(9):537-547 10.1136/bjsports-2022-106099 36889918

[2] Capogna BM Mahure SA Mollon B Duenes ML Rokito AS. Young age, female gender, Caucasian race, and workers’ compensation claim are risk factors for reoperation following arthroscopic ACL reconstruction. Knee Surg Sports Traumatol Arthrosc. 2020;28(7):2213-2223. 31813020 10.1007/s00167-019-05798-4

[3] Cavaignac E Coulin B Tscholl P Nik Mohd Fatmy N Duthon V Menetrey J . Is quadriceps tendon autograft a better choice than hamstring autograft for anterior cruciate ligament reconstruction? A comparative study with a mean follow-up of 3.6 years. Am J Sports Med. 2017;45(6):1326-1332. 28273424 10.1177/0363546516688665

[4] Collins NJ Prinsen CAC Christensen R Bartels EM Terwee CB Roos EM. Knee Injury and Osteoarthritis Outcome Score (KOOS): systematic review and meta-analysis of measurement properties. Osteoarthritis Cartilage. 2016;24(8):1317-1329. 27012756 10.1016/j.joca.2016.03.010

[5] Cristiani R Forssblad M Edman G Eriksson K Stålman A. Age, time from injury to surgery and quadriceps strength affect the risk of revision surgery after primary ACL reconstruction. Knee Surg Sports Traumatol Arthrosc. 2021;29(12):4154-4162. 33661322 10.1007/s00167-021-06517-8 PMC8595184

[6] Cristiani R Mikkelsen C Edman G Forssblad M Engström B Stålman A. Age, gender, quadriceps strength and hop test performance are the most important factors affecting the achievement of a patient-acceptable symptom state after ACL reconstruction. Knee Surg Sports Traumatol Arthrosc. 2020;28(2):369-380. 31230125 10.1007/s00167-019-05576-2 PMC6994649

[7] Dai W Leng X Wang J Cheng J Hu X Ao Y. Quadriceps tendon autograft versus bone–patellar tendon–bone and hamstring tendon autografts for anterior cruciate ligament reconstruction: a systematic review and meta-analysis. Am J Sports Med. 2022;50(12):3425-3439. 34494906 10.1177/03635465211030259

[8] Devana SK Solorzano C Nwachukwu BU Jones KJ. Disparities in ACL reconstruction: the influence of gender and race on incidence, treatment, and outcomes. Curr Rev Musculoskelet Med. 2022;15(1):1-9. 34970713 10.1007/s12178-021-09736-1 PMC8804118

[9] Ebert JR Calvert ND Radic R. A prospective randomized controlled trial investigating quadriceps versus hamstring tendon autograft in anterior cruciate ligament reconstruction. Am J Sports Med. 2024;52(3):660-669. 38284303 10.1177/03635465231222279 PMC10905979

[10] Ebert JR Calvert ND Radic R. Females demonstrate lower levels of activity, psychological readiness and strength symmetry after anterior cruciate ligament reconstruction than males, and also recovery of quadriceps strength and hop symmetry is delayed in females undergoing reconstruction with a quadriceps tendon autograft. Knee Surg Sports Traumatol Arthrosc. 2024;32(10):2688-2698. 39126259 10.1002/ksa.12426

[11] Ellison TM Flagstaff I Johnson AE. Sexual dimorphisms in anterior cruciate ligament injury: a current concepts review. Orthop J Sports Med. 2021;9(12):23259671211025304. 10.1177/23259671211025304 PMC8725014 34993256

[12] Hamrin Senorski E Svantesson E Baldari A , et al. Factors that affect patient reported outcome after anterior cruciate ligament reconstruction—a systematic review of the Scandinavian knee ligament registers. Br J Sports Med. 2019;53(7):410-417. 30030283 10.1136/bjsports-2017-098191

[13] Hansen CF Jensen J Odgaard A , et al. Four of five frequently used orthopedic PROMs possess inadequate content validity: a COSMIN evaluation of the mHHS, HAGOS, IKDC-SKF, KOOS and KNEES-ACL. Knee Surg Sports Traumatol Arthrosc. 2022;30(11):3602-3615. 34618175 10.1007/s00167-021-06761-y

[14] Hogan DW Burch MB Rund JM , et al. No difference in complication rates or patient-reported outcomes between bone–patella tendon–bone and quadriceps tendon autograft for anterior cruciate ligament reconstruction. Arthrosc Sports Med Rehabil. 2021;4(2):e417-e424. 10.1016/j.asmr.2021.10.019 PMC9042747 35494262

[15] Holmgren D Noory S Moström E Grindem H Stålman A Wörner T. Weaker quadriceps muscle strength with a quadriceps tendon graft compared with a patellar or hamstring tendon graft at 7 months after anterior cruciate ligament reconstruction. Am J Sports Med. 2024;52(1):69-76. 38164665 10.1177/03635465231209442 PMC10762885

[16] Hunnicutt JL Xerogeanes JW Tsai LC , et al. Terminal knee extension deficit and female sex predict poorer quadriceps strength following ACL reconstruction using all-soft tissue quadriceps tendon autografts. Knee Surg Sports Traumatol Arthrosc. 2021;29(9):3085-3095. 33175281 10.1007/s00167-020-06351-4

[17] Hurley ET Calvo-Gurry M Withers D Farrington SK Moran R Moran CJ. Quadriceps tendon autograft in anterior cruciate ligament reconstruction: a systematic review. Arthroscopy. 2018;34(5):1690-1698. 29628380 10.1016/j.arthro.2018.01.046

[18] Ingelsrud LH Granan LP Terwee CB Engebretsen L Roos EM. Proportion of patients reporting acceptable symptoms or treatment failure and their associated KOOS values at 6 to 24 months after anterior cruciate ligament reconstruction: a study from the Norwegian knee ligament registry. Am J Sports Med. 2015;43(8):1902-1907. 25977523 10.1177/0363546515584041

[19] Ingelsrud LH Terwee CB Terluin B , et al. Meaningful change scores in the knee injury and osteoarthritis outcome score in patients undergoing anterior cruciate ligament reconstruction. Am J Sports Med. 2018;46(5):1120-1128. 29517924 10.1177/0363546518759543

[20] Jackson GR Lee J Tuthill T , et al. Higher rates of residual postoperative instability after anterior cruciate ligament reconstruction in female patients: a systematic review of level II studies. Arthrosc Sports Med Rehabil. 2023;5(5). 10.1016/j.asmr.2023.100772 PMC10407150 37560145

[21] Kanakamedala AC de SA D Obioha OA , et al. No difference between full thickness and partial thickness quadriceps tendon autografts in anterior cruciate ligament reconstruction: a systematic review. Knee Surg Sports Traumatol Arthrosc. 2019;27(1):1795. 10.1007/s00167-018-5042-z 29974173

[22] Kvist J Kartus J Karlsson J Forssblad M. Results from the Swedish National Anterior Cruciate Ligament Register. Arthroscopy. 2014;30(7):803-810. 24746404 10.1016/j.arthro.2014.02.036

[23] Lin KM Boyle C Marom N Marx RG. Graft selection in anterior cruciate ligament reconstruction. Sports Med Arthrosc Rev. 2020;28(2):41-48. 32345925 10.1097/JSA.0000000000000265

[24] Lind M. Low surgical routine increases revision rates after quadriceps tendon autograft for anterior cruciate ligament reconstruction: results from the Danish knee ligament reconstruction registry. Knee Surg Sports Traumatol Arthrosc. 2021;29(6):1880-1886. 32886156 10.1007/s00167-020-06220-0

[25] Lind M Nielsen T. No difference in clinical outcome between quadriceps tendon anterior cruciate ligament reconstruction with and without bone block: results from the Danish Knee Ligament Registry. Knee Surg Sports Traumatol Arthrosc. 2025;33(5):1579-1585. 39302090 10.1002/ksa.12451 PMC12022824

[26] Lind M Nielsen TG Soerensen OG Mygind-Klavsen B Faunø P. Quadriceps tendon grafts does not cause patients to have inferior subjective outcome after anterior cruciate ligament (ACL) reconstruction than do hamstring grafts: a 2-year prospective randomised controlled trial. Br J Sports Med. 2020;54(3):183-187. 31704697 10.1136/bjsports-2019-101000

[27] Lind M Strauss MJ Nielsen T Engebretsen L. Quadriceps tendon autograft for anterior cruciate ligament reconstruction is associated with high revision rates: results from the Danish Knee Ligament Registry. Knee Surg Sports Traumatol Arthrosc. 2020;28(7):2163-2169. 31641810 10.1007/s00167-019-05751-5

[28] Maletis GB Funahashi TT Inacio MCS Paxton LW. Optimizing anterior cruciate ligament reconstruction: individualizing the decision-making process using data from the Kaiser Permanente ACLR registry: 2018 OREF award paper. J Orthop Res. 2022;40(1):29-42. 33751638 10.1002/jor.25020

[29] Mohtadi NG Chan DS. A randomized clinical trial comparing patellar tendon, hamstring tendon, and double-bundle ACL reconstructions: patient-reported and clinical outcomes at 5-year follow-up. J Bone Joint Surg Am. 2019;101(11):949-960. 31169571 10.2106/JBJS.18.01322

[30] Montalvo AM Schneider DK Yut L , et al. “What’s my risk of sustaining an ACL injury while playing sports?” A systematic review with meta-analysis. Br J Sports Med. 2019;53(16):1003-1012. 29514822 10.1136/bjsports-2016-096274 PMC6561829

[31] MOON Knee Group, Spindler KP Huston LJ , ,. Anterior cruciate ligament reconstruction in high school and college-aged athletes: does autograft choice influence anterior cruciate ligament revision rates? Am J Sports Med. 2020;48(2):298-309. 31917613 10.1177/0363546519892991 PMC7319140

[32] Mouarbes D Menetrey J Marot V Courtot L Berard E Cavaignac E . Anterior cruciate ligament reconstruction: a systematic review and meta-analysis of outcomes for quadriceps tendon autograft versus bone–patellar tendon-bone and hamstring-tendon autografts. Am J Sports Med. 2019;47(14):3531-3540. 30790526 10.1177/0363546518825340

[33] Prentice HA Lind M Mouton C , et al. Patient demographic and surgical characteristics in anterior cruciate ligament reconstruction: a description of registries from six countries. Br J Sports Med. 2018;52(11):716-722. 29574451 10.1136/bjsports-2017-098674

[34] Rahr-Wagner L Thillemann TM Pedersen AB Lind M. Comparison of hamstring tendon and patellar tendon grafts in anterior cruciate ligament reconstruction in a nationwide population-based cohort study: results from the Danish registry of knee ligament reconstruction. Am J Sports Med. 2014;42(2):278-284. 24275859 10.1177/0363546513509220

[35] Randsborg PH Cepeda N Adamec D Rodeo SA Ranawat A Pearle AD. Patient-reported outcome, return to sport, and revision rates 7-9 years after anterior cruciate ligament reconstruction: results from a cohort of 2042 patients. Am J Sports Med. 2022;50(2):423-432. 35040694 10.1177/03635465211060333 PMC8829731

[36] Reinholdsson J Kraus-Schmitz J Forssblad M Edman G Byttner M Stålman A. A non-response analysis of 2-year data in the Swedish Knee Ligament Register. Knee Surg Sports Traumatol Arthrosc. 2017;25(8):2481-2487. 26724828 10.1007/s00167-015-3969-x

[37] Rizvanovic D Waldén M Forssblad M Stålman A. Influence of surgeon experience and clinic volume on subjective knee function and revision rates in primary ACL reconstruction: a study from the Swedish National Knee Ligament Registry. Orthop J Sports Med. 2024;12(3):23259671241233695. 10.1177/23259671241233695 PMC10929050 38476163

[38] Rizvanovic D Waldén M Forssblad M Stålman A. Surgeon’s experience, sports participation and a concomitant MCL injury increase the use of patellar and quadriceps tendon grafts in primary ACL reconstruction: a nationwide registry study of 39,964 surgeries. Knee Surg Sports Traumatol Arthrosc. 2023;31(2):475-486. 35896755 10.1007/s00167-022-07057-5 PMC9898417

[39] Roos EM Boyle E Frobell RB Lohmander LS Ingelsrud LH. It is good to feel better, but better to feel good: whether a patient finds treatment “successful” or not depends on the questions researchers ask. Br J Sports Med. 2019;53(23):1474-1478. 31072841 10.1136/bjsports-2018-100260

[40] Roos EM Lohmander LS. The knee injury and osteoarthritis outcome score (KOOS): from joint injury to osteoarthritis. Health Qual Life Outcomes. 2003;1:64. 14613558 10.1186/1477-7525-1-64 PMC280702

[41] Runer A Csapo R Hepperger C Herbort M Hoser C Fink C. Anterior cruciate ligament reconstructions with quadriceps tendon autograft result in lower graft rupture rates but similar patient-reported outcomes as compared with hamstring tendon autograft: a comparison of 875 patients. Am J Sports Med. 2020;48(9):2195-2204. 32667271 10.1177/0363546520931829

[42] Runer A Wierer G Herbst E , et al. There is no difference between quadriceps- and hamstring tendon autografts in primary anterior cruciate ligament reconstruction: a 2-year patient-reported outcome study. Knee Surg Sports Traumatol Arthrosc. 2018;26(2):605-614. 28477270 10.1007/s00167-017-4554-2

[43] Ryan J Magnussen RA Cox CL Hurbanek JG Flanigan DC Kaeding CC. ACL reconstruction: do outcomes differ by sex? A systematic review. J Bone Jt Surg Am. 2014;96(6):507-512. 10.2106/JBJS.M.00299 24647508

[44] Salminen M Kraeutler MJ Freedman KB , et al. Choosing a graft for anterior cruciate ligament reconstruction: surgeon influence reigns supreme. Am J Orthop. 2016;45(4):192-197. 27327925

[45] Shani RH Umpierez E Nasert M Hiza EA Xerogeanes J. Biomechanical comparison of quadriceps and patellar tendon grafts in anterior cruciate ligament reconstruction. Arthroscopy. 2016;32(1):71-75. 26382635 10.1016/j.arthro.2015.06.051

[46] Slone HS Romine SE Premkumar A Xerogeanes JW. Quadriceps tendon autograft for anterior cruciate ligament reconstruction: a comprehensive review of current literature and systematic review of clinical results. Arthroscopy. 2015;31(3):541-554. 25543249 10.1016/j.arthro.2014.11.010

[47] Svantesson E Hamrin Senorski E Baldari A , et al. Factors associated with additional anterior cruciate ligament reconstruction and register comparison: a systematic review on the Scandinavian knee ligament registers. Br J Sports Med. 2019;53(7):418-425. 30018121 10.1136/bjsports-2017-098192

[48] Tan SHS Lau BPH Khin LW Lingaraj K . The importance of patient sex in the outcomes of anterior cruciate ligament reconstructions: a systematic review and meta-analysis. Am J Sports Med. 2016;44(1):242-254. 25802119 10.1177/0363546515573008

[49] Vascellari A Grassi A Canata GL Zaffagnini S Gokeler A Jones H. Hamstrings substitution via anteromedial portal with optional anterolateral ligament reconstruction is the preferred surgical technique for anterior cruciate ligament reconstruction: a survey among ESSKA members. Knee Surg Sports Traumatol Arthrosc. 2021;29(4):1120-1127. 32591846 10.1007/s00167-020-06107-0

[50] White T Castro M Antonio L Hing W Tudor F Sattler L . Quadriceps, hamstring and patella tendon autografts for primary anterior cruciate ligament reconstruction demonstrate similar clinical outcomes, including graft failure, joint laxity and complications: a systematic review with meta-analysis of randomised controlled trials. Knee Surg Sports Traumatol Arthrosc. 2025; Epub ahead of print. doi.org/10.1002/ksa.12755 10.1002/ksa.12755 PMC13122740 40679231

[51] Zegzdryn M Moatshe G Engebretsen L , et al. Increased risk for early revision with quadriceps graft compared with patellar tendon graft in primary ACL reconstructions. Knee Surg Sports Traumatol Arthrosc. 2024;32(3):656-665. 38375583 10.1002/ksa.12081

[52] Zhou Y Fuimaono-Asafo A Frampton C van Niekerk M Hirner M. Quadriceps tendon autograft is comparable to hamstring tendon and bone-patella-tendon-bone up to 2 years after isolated primary anterior cruciate ligament reconstruction. Knee Surg Sports Traumatol Arthrosc. 2023;31(8):1795. 10.1007/s00167-023-07370-7 36894784

